# Case report: Frontotemporal dementia and amyotrophic lateral sclerosis caused by a missense variant (p.Arg89Trp) in the valosin-containing protein gene

**DOI:** 10.3389/fgene.2023.1155998

**Published:** 2023-05-26

**Authors:** Shiroh Miura, Shigeyoshi Hiruki, Tomohisa Okada, Satoko Itani Takei, Kensuke Senzaki, Yoko Okada, Masayuki Ochi, Yuki Tanabe, Hirofumi Ochi, Michiya Igase, Yasumasa Ohyagi, Hiroki Shibata

**Affiliations:** ^1^ Department of Neurology and Geriatric Medicine, Ehime University Graduate School of Medicine, Toon, Ehime, Japan; ^2^ Division of Genomics, Medical Institute of Bioregulation, Kyushu University, Higashi-ku, Fukuoka, Japan; ^3^ Department of Radiology, Ehime University Graduate School of Medicine, Toon, Ehime, Japan; ^4^ Department of Intractable Disease and Aging Science, Ehime University Graduate School of Medicine, Toon, Ehime, Japan; ^5^ Department of Anti-aging Medicine, Ehime University Graduate School of Medicine, Toon, Ehime, Japan

**Keywords:** frontotemporal dementia and/or amyotrophic lateral sclerosis-6 (FTDALS6), amyotrophic lateral sclerosis 14 (ALS14), VCP, cerebellar atrophy, missense variant

## Abstract

Frontotemporal dementia and/or amyotrophic lateral sclerosis 6, also known as amyotrophic lateral sclerosis 14, is an autosomal dominant, progressive neurodegenerative disorder caused by various mutations in the valosin-containing protein gene. In this report, we examined a 51-year-old female Japanese patient with frontotemporal dementia and amyotrophic lateral sclerosis. The patient began noticing gait disturbances at the age of 45 years. Neurological examination at the age of 46 years met the Awaji criteria for clinically probable amyotrophic lateral sclerosis. At the age of 49 years, she tended to have poor mood and an aversion to activity. Her symptoms gradually worsened. She required a wheelchair for transport and had difficulty communicating with others because of poor comprehension. She then began to frequently exhibit irritability. Eventually, she was admitted to the psychiatric hospital because uncontrollable violent behavior throughout the day. Longitudinal brain magnetic resonance imaging revealed progressive brain atrophy with temporal dominance, non-progressive cerebellar atrophy, and some non-specific white matter intensities. Brain single photon emission computed tomography showed hypoperfusion in the bilateral temporal lobes and cerebellar hemispheres. Clinical exome sequencing revealed the presence of a heterozygous nonsynonymous variant (NM_007126.5, c.265C>T; p.Arg89Trp) in the valosin-containing protein gene, which was absent in the 1000 Genomes Project, the Exome Aggregation Consortium Database, and the Genome Aggregation Database, and was predicted to be “damaging” by PolyPhen-2 and “deleterious” using SIFT with a Combined Annotation Dependent Depletion score of 35. We also confirmed the absence of this variant in 505 Japanese control subjects. Therefore, we concluded that the variant in the valosin-containing protein gene was responsible for the symptoms of this patient.

## Introduction

The valosin-containing protein (*VCP*, MIM: 601023) gene is considered responsible for frontotemporal dementia (FTD) and/or amyotrophic lateral sclerosis (ALS) 6 (FTDALS6), also known as ALS14 (MIM: 613954), and inclusion body myopathy with early-onset Paget disease and frontotemporal dementia 1 (MIM: 167320) via a dominant-negative mechanism (Ayaki et al., 2014). It is known to exhibit large intrafamilial and interfamilial phenotypic variations among patients with *VCP* mutations (Al-Obeidi et al., 2018). Myopathy, Paget disease of bone (PDB), FTD, ALS, Parkinson’s disease, and Alzheimer’s disease occur in 89.8%–91.0%, 42.4%–51.7%, 29.6%–30.3%, 8.6%–8.9%, 3.4%–3.8%, and 1.6% of patients carrying *VCP* mutations, respectively (Al-Obeidi et al., 2018; Mehta et al., 2013). Regarding ALS, the frequency of *VCP* mutations in familial ALS patients, young-onset ALS patients (age of onset ≤45), and sporadic ALS patients are up to 2%, 3.7%, and 0.4%, respectively (González-Pérez et al., 2012; Johnson et al., 2010; Koppers et al., 2012; Deng et al., 2019; Abramzon et al., 2012). For FTD, 3.5% of patients carry *VCP* mutations (Saracino et al., 2018). The coexistence of ALS and FTD (ALS-FTD) is a rare manifestation among patients carrying *VCP* mutations. Here, we report a Japanese patient with ALS-FTD carrying a missense variant of the *VCP* gene. We also present brain magnetic resonance imaging (MRI) findings over time to demonstrate disease progression.

### Clinical report

A 51-year-old woman, who had no family history of neuropsychiatric diseases, began noticing gait disturbance at the age of 45 years and upper limb weakness at the age of 46 years. These symptoms gradually worsened. Her personality showed a mild change. She developed depression associated with obsessive behavior and difficulty understanding everyday speech at the age of 49 years. She began using a wheelchair for transport at the age of 50 years. Finally, she was admitted to a psychiatric hospital because she was easily distracted and behaved violently throughout the day. Her past medical history revealed a transient ischemic attack at the age of 41 years. Neurological examination at the age of 46 years showed that the patient had mild muscular weakness of the four limbs with left distal dominance. Amyotrophy was unremarkable. Her hand grip was 18 kg in the right hand and 11 kg in the left hand. She showed hyperreflexia and spasticity in all four extremities with left dominance, and her jaw reflex was increased. Furthermore, her Babinski reflexes were positive bilaterally, and her gait was spastic. There were no abnormalities in the cranial nerves. Sensory, autonomic, and cerebellar functions were normal. Nerve conduction studies revealed that F-wave occurrences were reduced in the median nerve bilaterally (right: 56%, left: 25%; normal values: 70% <). Amplitudes of compound muscle action potentials, motor conduction velocities, amplitudes of sensory nerve action potentials, and sensory conduction velocities were normal in all nerves tested (i.e., the bilateral median, ulnar, tibial, and sural nerves). Neurogenic changes were detected in the biceps brachii muscle, the first interossei dorsalis muscle, the thoracic paraspinal muscle, the quadriceps femoris muscle, and the tibialis anterior muscle using needle electromyography. Upon neurological examination at the age of 50 years, the patient showed attention disturbances, transcortical sensory aphasia, and a positive snout reflex. Hyperreflexia, spasticity, and weakness in all four extremities had worsened, and bilateral ankle contracture was observed. She was unable to maintain a standing position. Her hand grip was 16 kg in the right hand and 6 kg in the left hand. There were no abnormalities in the cranial nerves or sensory, autonomic, and cerebellar functions. Her Mini-Mental State Examination score was 16/30, Frontal Assessment Battery score was 5/18, Alzheimer’s Disease Assessment Scale was 29.3/70, and Raven’s Colored Progressive Matrices was 32/36.

**FIGURE 1:**
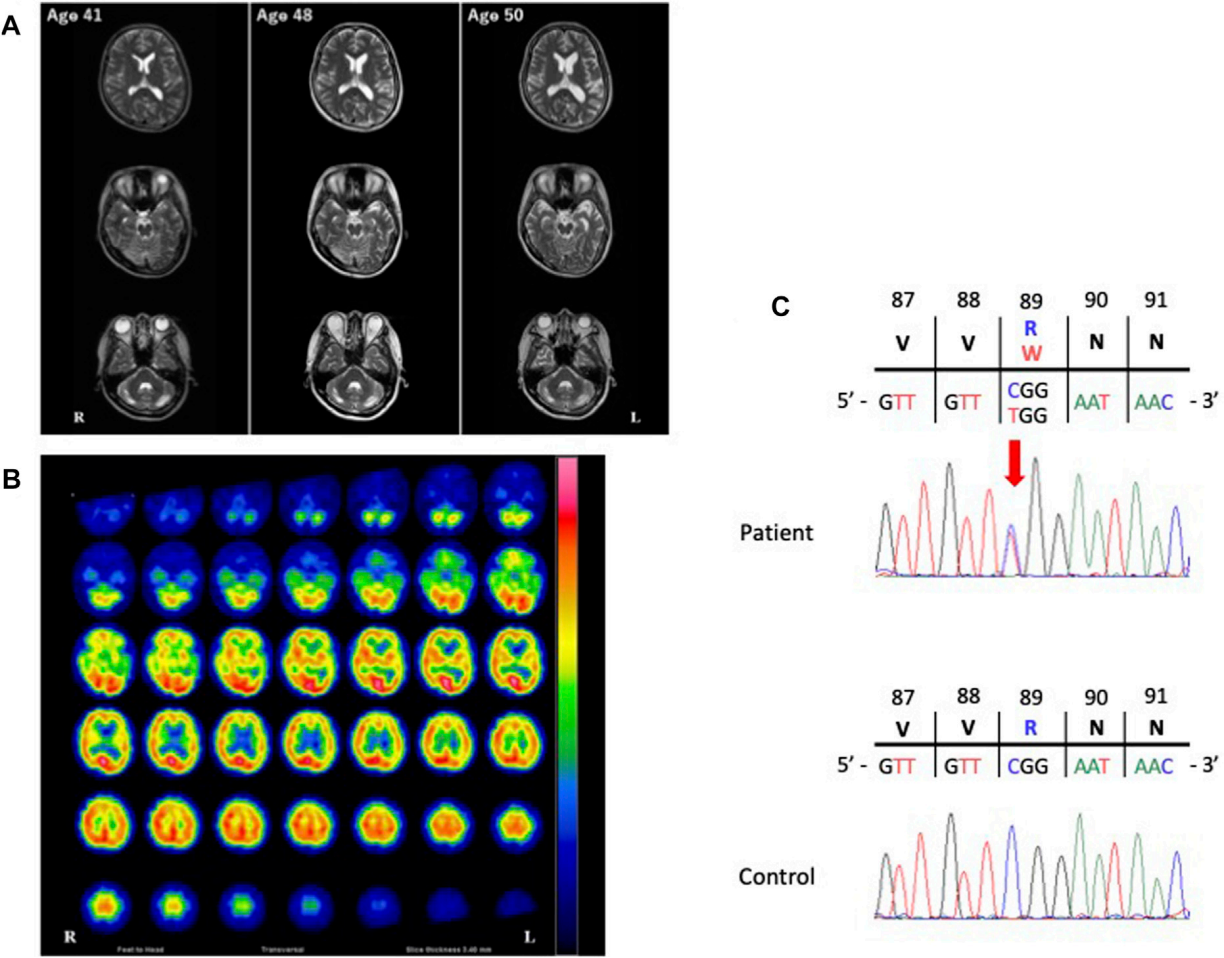
Images of the present patient and the nucleotide variant. **(A)**, The course of T2-weighted brain magnetic resonance imaging (MRI). Longitudinal brain MRI showed progressive brain atrophy with left temporal dominance and non-progressive cerebellar atrophy. **(B)**, Brain N-isopropyl-p-[123I] iodoamphetamine single photon emission computed tomography (^123^I-IMP SPECT). The patient’s^123^I-IMP SPECT image acquired at the age of 50 years showed hypoperfusion in the bilateral temporal lobes with left dominance and the bilateral cerebellar hemispheres. **(C)** Electropherogram of the region of the variant of the valosin-containing protein gene (NM_007126.5: exon 9: c.265C>T [Arg89Trp]) in our patient and an unaffected control. The location of the variant is indicated by a red arrow.

The cerebrospinal fluid examination was negative, as were the test results for oligoclonal bands and immunoglobulin G indices at the ages of 41, 46, 48, and 50 years. Furthermore, the levels of serum creatine kinase and alkaline phosphatase were normal throughout her clinical course. Longitudinal brain MRI revealed progressive brain atrophy with temporal dominance (Figure 1A), non-progressive cerebellar atrophy (Figure 1A), and some non-specific white matter intensities (data not shown). Brain N-isopropyl-p-[123I] iodoamphetamine single photon emission computed tomography (SPECT) at the age of 50 years showed hypoperfusion in the bilateral temporal lobe with left dominance and the cerebellar hemispheres (Figure 1B). Neither the cervical MRI acquired at the age of 41 years nor the whole spinal MRI acquired at the age of 46 years showed abnormalities.

Preliminary genetic analyses confirmed the absence of repeat expansions in 11 genes known to be associated with cerebellar atrophy: *ATXN1*, *ATXN2*, *ATXN3*, *CACNA1A*, *ATXN7*, *ATXN8*, *ATXN10*, *PPP2R2B*, *TBP*, *NOP56,* and *ATN1*.

Her clinical or electrophysiological findings showed upper and lower motor neuron signs which met clinically probable ALS of the Awaji criteria (Table 1). Also, she did not exhibit any clinical or laboratory findings suggestive of myopathy or PDB throughout her clinical course.

**TABLE 1:** Distribution of lower and upper motor neuron signs in the present case.

—	Bulbar	Cervical	Thoracic	Lumbosacral
Lower motor neuron signs	-	+	+	+
Upper motor neuron signs	+	+	-	+

Clinically probable ALS, is defined on clinical or electrophysiological evidence by lower motor neuron and upper motor neuron signs in at least two regions with some upper motor neuron signs necessarily rostral to the lower motor neuron signs.

## Materials and methods

### Patient information

The patient was Japanese and originated in Ehime Prefecture of Shikoku Island in Japan. A peripheral blood sample was collected. Unfortunately, peripheral blood samples were unavailable from any other family members due to the private reasons.

### Whole-exome sequencing

We performed whole-exome sequencing. Genomic DNA was extracted from the peripheral blood using a QIAamp DNA Blood Kit (Qiagen, Hilden, Germany). Exonic regions were enriched using SureSelect Human All Exon v6 (Agilent Technologies, Santa Clara, CA, United States). Paired-end sequencing at 150 bp was performed using the Illumina NovaSeq6000 platform (Illumina, Inc., CA, United States). Raw sequencing reads were analyzed using Trimmomatic v0.36, BWA v0.7.16, Samtools v1.5, and Genome Analysis Toolkit v3.8.0. Variants were annotated using wAnnovar (https://wannovar.wglab.org/).

Because of cerebellar atrophy and the ALS symptoms exhibited by the patient, we prioritized the analysis of variants located in genes 77 and 72, which are known to be associated with hereditary spinocerebellar ataxias (SCAs) and hereditary ALSs, respectively (Supplementary Table S1). We examined the frequencies of candidate variants in control populations using the 1000 Genomes Project (1000G; http://www.1000genomes.org), the Exome Aggregation Consortium (ExAC) database (http://exac.broadinstitute.org), the Genome Aggregation Database (gnomAD; https://gnomad.broadinstitute.org/) and the Japanese Multi-Omics Reference Panel (jMorp; https://jmorp.megabank.tohoku.ac.jp). We further removed non-pathogenic local variants found in the in-house exome data of 56 unrelated Japanese individuals who were matched geographically. The functional consequences of the variants were evaluated *in silico* using PolyPhen-2 (http://genetics.bwh.harvard.edu/pph2/), SIFT (https://sift.bii.a-star.edu.sg/), and Combined Annotation Dependent Depletion (CADD; http://cadd.gs.washington.edu/home).

### Sanger sequencing

The region that included the variation site in exon 9 of the *VCP* gene was amplified using the following primers: 5′-TTT​CTT​TCC​TCA​GCC​CAA​GA-3′ (forward) and 5′-ATC​GAC​AGG​TGC​CAA​GAA​CT-3′ (reverse). Polymerase chain reaction (PCR) conditions were 35 cycles of 94°C for 30 s, 58°C for 30 s, and 72°C for 30 s. PCR products were sequenced using the ABI PRISM Big Dye Terminator (v 3.1) Cycle Sequencing Kit (Thermo Fisher Scientific, Waltham, MA, United States) and the ABI PRISM 3130-Avant Genetic Analyzer (Thermo Fisher Scientific, Waltham, MA, United States).

## Results

### Exome sequencing

A mean read depth of 106.5× was achieved for the target regions. From 23,766 variants initially called, we identified 75 single nucleotide variants (SNVs) located in genes known to be responsible for hereditary SCAs (Supplementary Table S1). None of the SNVs met our filtering criteria of a minor allele frequency of <0.001 in the public databases. We also identified 74 SNVs located in genes known to be responsible for hereditary ALSs (Supplementary Table S1), which comprised 32 homozygous and 42 heterozygous variants. Of the 74 variants, two SNVs met the criteria of a minor allele frequency of <0.001 in the public databases. We further excluded one of these variants, which was predicted to be “benign” and “tolerated” by PolyPhen-2 and SIFT, respectively. Thus, only one heterozygous non-synonymous variant located on exon 9 of the *VCP* gene was retained (NM_007126.5, c.265C>T [Arg89Trp]). This variant was confirmed to be absent in the exome data of 56 unrelated in-house controls.

### Validation of the VCP variant using Sanger sequencing

The *VCP* variant (NM_007126.5, c.265C>T [Arg89Trp]) was absent in 1000G, ExAC, gnomAD and jMorp, and was predicted to be “damaging” by PolyPhen-2 and “deleterious” by SIFT, with a CADD score of 35 (Figure 1C). We further confirmed the absence of the variant in 505 healthy, unrelated Japanese individuals using Sanger sequencing, which indicated that the variant is extremely rare in the Japanese population, with a frequency of <0.001.

## Discussion

We described a Japanese FTDALS6 case carrying a rare variant (c.265C>T) in the *VCP* gene that resulted in an amino acid substitution (Arg89Trp). The variant has been registered in ClinVar with no clinical information (Allele ID: 993443). The patient’s neurological findings at the age of 46 years met the Awaji ALS criteria for clinically probable ALS (de Carvalho et al., 2008). Subsequently, a diagnosis of ALS complicated with FTD was made according to the revised diagnostic criteria (Strong et al., 2017). To date, at least 10 cases of ALS complicated with FTD have been reported to be associated with *VCP* variants (Miller et al., 2009; Kumar et al., 2010; Johnson et al., 2010; Koppers et al., 2012; Spina et al., 2013; Hirano et al., 2015; Hirano et al., 2017; Matsubara et al., 2021). Clinical and/or pathological information of previously reported patients with ALS complicated with FTD carrying *VCP* variants are summarized in Table 2, which includes our patient. In most cases, the initial symptom of ALS preceded that of FTD. Six out of 11 cases showed no neurological signs except for ALS and FTD. Other neurological signs observed were myopathy (two cases), hearing impairment (two cases), PDB (two cases), parkinsonism (one case), and urinary urge incontinence (one case). Neither serum alkaline phosphatase nor creatine kinase was elevated in most cases. Moreover, no specific variants were correlated with the incidence of clinical features of ALS complicated with FTD. In the current case, as well as previously reported cases, no abnormalities were observed on brain MRI before the onset of dementia and/or psychotic symptoms (Johnson et al., 2010; Matsubara et al., 2021). However, once dementia and/or psychotic symptoms appeared, frontotemporal atrophy seemed to progress. This phenomenon is compatible with the fact that ALS usually precedes FTD, although one report showed that modest temporal atrophy was observed before the onset of dementia-associated symptoms (Hirano et al., 2017). In general, frontotemporal atrophy has been shown to progress as dementia and/or psychotic symptoms worsen. However, the degree of brain atrophy varies among individuals with the same symptom severity. In addition, some non-specific cerebral white matter intensities were associated with the disease. Indeed, SPECT examinations have revealed that hypoperfusion in the left temporal lobe may be a distinct feature of the disease.

**TABLE 2:** Clinical characteristics of the present case and previously reported cases with amyotrophic lateral sclerosis plus frontotemporal dementia with valosin-containing protein variants.

—	Case 1	Case 2	Case 3	Case 4	Case 5	Case 6	Case 7	Case 8	Case 9	Case 10	—
Year	2009	2010	2010	2010	2012	2013	2013	2015/2017	2017	2021	Our case
Author	Miller et al.	Kumar et al.	Johnson et al.	Johnson et al.	Koppers et al.	Spina et al.	Spina et al.	Hirano et al./Hirano et al.	Hirano et al.	Matsubara et al.	—
Site of variant	NM_007126.5, c.464G>A [p.Arg155His]	NM_007126.5, c.464G>T [p.Arg155Leu]	NM_007126.5, c.572G>A [p.Arg191Gln]	NM_007126.5, c.475C>G [p.Arg159Gly]	NM_007126.5, c.476G>A [p.Arg159His]	NM_007126.5, c.572G>A [p.Arg191Gln]	NM_007126.5, c.784A>G [p.Thr262Ala]	NM_007126.5, c.1460G>A [p.Arg487His]	NM_007126.5, c.1460G>A [p.Arg487His]	NM_007126.5, c.1460G>A [p.Arg487His]	NM_007126.5, c.265C>T [p.Arg89Trp]
Origin	British	Australian	Italian	United States	Netherlander	United States	United States	Japanese	Japanese	Japanese?	Japanese
Initial symptom	NA	difficulty climbing stairs, getting out of chairs and getting up off the floor	fasciculations and cramping of the right leg	leg weakness with difficulty walking	weakness in hands	stiffness and cramps in arms and legs	forgetting names	bilateral weakness of the proximal upper limbs	difficulty singing with a high pitch	stiffness in the left leg	gait disturbance
Age at onset of ALS (yr)	49	early forties	50	53	59	63	53	61	73	62	45
Age at onset of FTD (yr)	NA	late forties	50?	50?	NA (diagnosed by autopsy)	after 65	51	70	74?	65	49
Family history for ALS + FTD	+?	-	+?	-	+	-	-	-	-	-	-
Mini-Mental State examination score	NA	NA	NA	NA	NA	NA	NA	NA	21/30at the age of 74	21/30 at the age of 64	16/30 at the age of 50
Frontal Assessment Battery score	NA	NA	14/18 at the age of 51	NA	NA	NA	NA	NA	6/18 at the age of 74, 3/18 at the age of 75	15/18 at the age of 64	5/18 at the age of 50
Addenbrooke’s Cognitive Examination - Revised score	67/100, frontal pattern	NA	NA	NA	NA	NA	NA	NA	NA	NA	not examined
Other clinical signs along disease course	moderate left ventricle function which appears stiff., mild mitral regurgitation, urinary urge incontinence, PDB	myopathy, PDB, sensorineural hearing loss	none	none	none	myopathy, parkinsonism	terrible weight gain, poor sleep	cervical laminectomy, hearing impairments (no apparent response of wave I in auditory brainstem response test)	none	none	none
Elevated serum alkaline phosphatase level	-	+	-	NA	NA	NA	NA	slightly elevated, but the bone-specific alkaline phosphatase level was normal	elevated, but the bone-specific alkaline phosphatase level was normal	-	-
Elevated serum creatine kinase level	-	+	+	NA	NA	NA	NA	-	-	-	-
Brain MRI	asymmetrical left frontotemporal atrophy, scattered cerebral white matter intensities	normal	normal	NA	NA	NA	NA	marked atrophy of the frontal and temporal lobes	mildly progressive atrophy of the frontal and temporal lobes (followed by computed tomography images)	normal at the age of 62 (Mild atrophy involving the frontal lobe and precentral gyrus in autopsy at the age of 68)	progressive brain atrophy with left temporal dominancy, non-progressive cerebellar atrophy, some nonspecific cerebral white matter intensities
Spinal MRI	atrophic cord especially around the conus	normal	normal	NA	NA	NA	NA	NA	NA	normal	normal
Hypoperfusion on SPECT	NA	NA	NA	NA	NA	NA	left temporal lobe	NA	NA	NA	bilateral temporal lobe with left dominancy, bilateral cerebellar hemispheres

MRI, magnetic resonance imaging; SPECT, single photon emission computed tomography; ALS, amyotrophic lateral sclerosis; FTD, frontotemporal dementia; PDB, Paget’s disease of the bone; NA, not available.

Although we examined variants in the genes known to be associated with hereditary SCAs based on the cerebellar atrophy observed in the current case, no functional variant survived our filtering criteria. Moreover, there was neither progression of cerebellar atrophy nor cerebellar symptoms. Thus, we concluded that the patient’s cerebellar atrophy was either congenital or in a premature state. The association between cerebellar atrophy and the variant in the *VCP* gene remains unclear.

Table 3 summarizes the patients with missense variants at residue 89 in the *VCP* gene. The p.Arg89Trp variant has been previously reported in one Portuguese patient with distal myopathy and FTD (de Campos and de Carvalho, 2019). Another missense variant at the same residue (NM_007126.5, c.266G>A; p.Arg89Gln) in the *VCP* gene has been reported in a Chinese patient with young-onset ALS, who died of respiratory failure 5 months after the onset of initial symptoms (Deng et al., 2019). The clinical features varied among the three cases. Thus, there is no correlation between clinical phenotype and variants at residue 89 in the *VCP* gene. According to the American College of Medical Genetics and Genomics (ACMG)/Association for Molecular Pathology (AMP)/College of American Pathologists (CAP) guidelines, the non-synonymous variant in the *VCP* gene is classified as “pathogenic” because it meets the criteria of PS1, PM1, and PM2 (Richards et al., 2015). Therefore, we concluded that the missense variant in the *VCP* gene, NM_007126.5, c.265C>T; p.Arg89Trp, is the variant that causes the disease. Accordingly, we highlight the importance of examining *VCP* variations in patients with ALS and FTD, even in those with no family history of these diseases.

**TABLE 3:** Summary of missense variants reported at residue 89 in the valosin-containing protein gene.

—	Case 1	Case 2	—
Year	2019	2019	Our case
Author	de Campos et al.	Deng et al.	—
Site of variant	NM_007126.5, c.265C>T [p.Arg89Trp]	NM_007126.5, c.266G>A [p.Arg89Gln]	NM_007126.5, c.265C>T [p.Arg89Trp]
Origin	Portuguese	Chinese	Japanese
Age at onset (yr)	54	24	45
Initial symptom	symmetric distal upper limb weakness and atrophy	limb weakness	gait disturbance
Phenotype	distal myopathy, FTD	ALS	ALS, FTD
Elevated serum alkaline phosphatase level	—	NA	—
Elevated serum creatine kinase level	—	NA	—
Brain MRI	diffuse severe cortico-subcortical brain atrophy	NA	progressive brain atrophy with left temporal dominancy, non-progressive cerebellar atrophy, some nonspecific cerebral white matter intensities
Spinal MRI	NA	NA	normal
Hypoperfusion on SPECT	NA	NA	bilateral temporal lobe with left dominancy, bilateral cerebellar hemispheres
Hypometabolism on FDG-PET	bilateral frontal and anterior temporal lobes	NA	NA

MRI, magnetic resonance imaging; SPECT, single photon emission computed tomography; FDG-PET, 18F-fluorodeoxyglucose-positron emission tomography; ALS, amyotrophic lateral sclerosis; FTD, frontotemporal dementia; IBM, inclusion body myopathy; PDB, Paget’s disease of the bone; NA, not available.

## Data Availability

The datasets for this article are not publicly available due to concerns regarding participant/patient anonymity. Requests to access the datasets should be directed to the corresponding author.
